# Species complex delimitations in the genus *Hedychium*: A machine learning approach for cluster discovery

**DOI:** 10.1002/aps3.11377

**Published:** 2020-07-31

**Authors:** Preeti Saryan, Shubham Gupta, Vinita Gowda

**Affiliations:** ^1^ Department of Biological Sciences Indian Institute of Science Education and Research Bhopal Bhopal Bypass Road Bhopal Madhya Pradesh 462066 India; ^2^ Department of Computer Science and Automation Indian Institute of Science Bengaluru Karnataka 560012 India

**Keywords:** cluster characterization, *Hedychium*, morphological analysis, spectral clustering

## Abstract

**Premise:**

Statistical methods used by most morphologists to validate species boundaries (such as principal component analysis [PCA] and non‐metric multidimensional scaling [nMDS]) are limiting because these methods are mostly used as visualization methods, and because the groups are identified by taxonomists (i.e., supervised), adding human bias. Here, we use a spectral clustering algorithm for the unsupervised discovery of species boundaries followed by the analysis of the cluster‐defining characters.

**Methods:**

We used spectral clustering, nMDS, and PCA on 16 morphological characters within the genus *Hedychium* to group 93 individuals from 10 taxa. A radial basis function kernel was used for the spectral clustering with user‐specified tuning values (gamma). The goodness of the discovered clusters using each gamma value was quantified using eigengap, a normalized mutual information score, and the Rand index. Finally, mutual information–based character selection and a *t*‐test were used to identify cluster‐defining characters.

**Results:**

Spectral clustering revealed five, nine, and 12 clusters of taxa in the species complexes examined here. Character selection identified at least four characters that defined these clusters.

**Discussion:**

Together with our proposed character analysis methods, spectral clustering enabled the unsupervised discovery of species boundaries along with an explanation of their biological significance. Our results suggest that spectral clustering combined with a character selection analysis can enhance morphometric analyses and is superior to current clustering methods for species delimitation.

Classification or categorization is the process by which objects are sorted based on a set of characters. In biology, the utility of classification is quite apparent because the fundamental unit that describes any organism is that of a “species,” which is expected to be governed by a set of characters that uniquely identifies it (see synapomorphy; de Queiroz, 2007). However, classification or categorization need not be limited to taxonomic or phylogenetic studies; they can be extended to any science where a set of characters are used to classify objects into distinct categories, such as in metagenomics, biodiversity characterization, character evolution, population genetics, ecological studies, and conservation (Sites and Crandall, 1997; Agapow et al., 2004; Cutler et al., 2007; Bennett and Balick, 2014; Vervier et al., 2016).

In biology, conceptualizing a universal definition of “species” has been challenging (Balakrishnan, 2005; de Queiroz, 2007). In macroorganisms, for all practical purposes, species are still linked to morphological character descriptions, which are often coded by the researcher using various tools such as imaging, character state, and qualitative and quantitative measurements. These characters are then subjected to statistical analyses whose purpose is to validate the species boundaries that have been set by our innate perception based on the morphological similarities between the organisms being studied. One of the taxonomic problems for which statistical approaches are critical is the resolution of taxonomic boundaries within a species complex. A species complex is defined as a group of taxa that pose a challenge in their identification because they have morphological characteristics that are very similar to other closely affiliated taxa. Traditionally, the grouping of individuals (by similarity) is often done using ordination methods, such as principal component analysis (PCA) or non‐metric multidimensional scaling (nMDS), which are unsupervised statistical approaches; however, these analyses are used for the supervised classification of groups rather than the unsupervised discovery of clusters (which is a distinction between classification and clustering, explained next).

With the advancement in computational power and high‐throughput methods, many other robust clustering‐based unsupervised learning methods are now available that can provide novel approaches to categorize the taxa that form species complexes (Kelley and Salzberg, 2010; Gisbrecht et al., 2013). Machine learning methods have most recently been used in computationally intensive biological fields such as genome‐level studies or bioinformatic investigations (Kelley and Salzberg, 2010; Gisbrecht et al., 2013), but their use in plant sciences is limited and mostly restricted to image analysis (Singh et al., 2016 and the references within; Pound et al., 2017). In this case, these methods have been primarily used for classification (such as support vector machine; Singh et al., 2016) rather than for identification of de novo clusters. This may be because the machine learning tools (especially for clustering) are not well documented, easily executable, or easily relatable, and they are mostly computationally intensive and data‐hungry (i.e., require large training data sets).

Classification and clustering sensu stricto do not refer to the same process. When performing classification, an annotated “ground truth” label for each sample is required, whereas clustering is concerned with the unsupervised discovery of patterns in data. Both these methods are commonly employed in the analysis of morphological and molecular data to delimit species boundaries (Cutler et al., 2007; McDonnell et al., 2019). Classification‐based approaches (such as discriminant analysis or random forest) are supervised because they require the user to annotate each individual with a category label (Cutler et al., 2007). In such methods, there is no scope for a post‐hoc exploratory analysis that might reveal insights not captured by the initial analysis used to identify the categories. Furthermore, the results from classification methods that require manual identification may be less accurate simply because of an inadequate level of domain expertise in the system (Culverhouse et al., 2003). Ordination methods such as PCA are unsupervised, but their use in biology is rather simplified (Dollhopf et al., 2001; McDonnell et al., 2019). In these analyses, data are projected into a lower‐dimensional space. The data points are identified based on the ground truth labels, and sometimes a convex hull is drawn around the points that share common labels, enabling the user to visualize the boundaries. The overlap among these convex hulls is then used as an indicator of the statistical goodness of the proposed species delimitations. Hence, inferences made purely based on ground truth labels and/or convex hull overlap only validate the species delimitations already proposed by human intervention, resulting in an almost circular argument toward species delimitation.

In contrast, unsupervised approaches (such as PCA, nMDS, and clustering methods), when used appropriately, allow the discovery of clusters by potentially uncovering or exploring critical features within the data that may have been otherwise overlooked due to human bias (Van Beuningen and Busch, 1997; Ezard et al., 2010). From a clustering perspective, transformations such as PCA and nMDS may not be very useful because clusters that are non‐convex in the input space may still remain non‐convex in the projected data space. As *k*‐means looks for convex, spherical clusters, it tends to perform better in conjunction with the transformation method used in spectral clustering. Spectral clustering is good at partitioning the data, given an appropriate similarity matrix, even in cases where the convex hulls corresponding to the clusters overlap prior to transformation. The spectral clustering algorithm is also a highly efficient, robust, streamlined, and automated method that improves the process in which clusters are discovered.

Here, we present a case from the genus *Hedychium* J. Koenig (Zingiberaceae), in which the morphological similarity between species is known to be very high, resulting in many species complexes that pose a challenge in species delimitation. We specifically use two species complexes as exemplar data sets, which are subjected to existing statistical tools such as PCA and nMDS for delimiting species within the complexes. We discuss the drawbacks of these tools in delimiting species and argue that spectral clustering (von Luxburg, 2007), a machine learning algorithm, can efficiently delimit species based on morphology. We chose to use spectral clustering as it can discover arbitrarily shaped clusters using an appropriate similarity measure, as opposed to the simplistic and more commonly used *k*‐means clustering algorithm, which can only discover spherical clusters. We also present statistical methods that can be used for feature selection, which translates to the identification of morphological characters that define each cluster in the analyses.

## METHODS

### Study species

**Figure 1:**
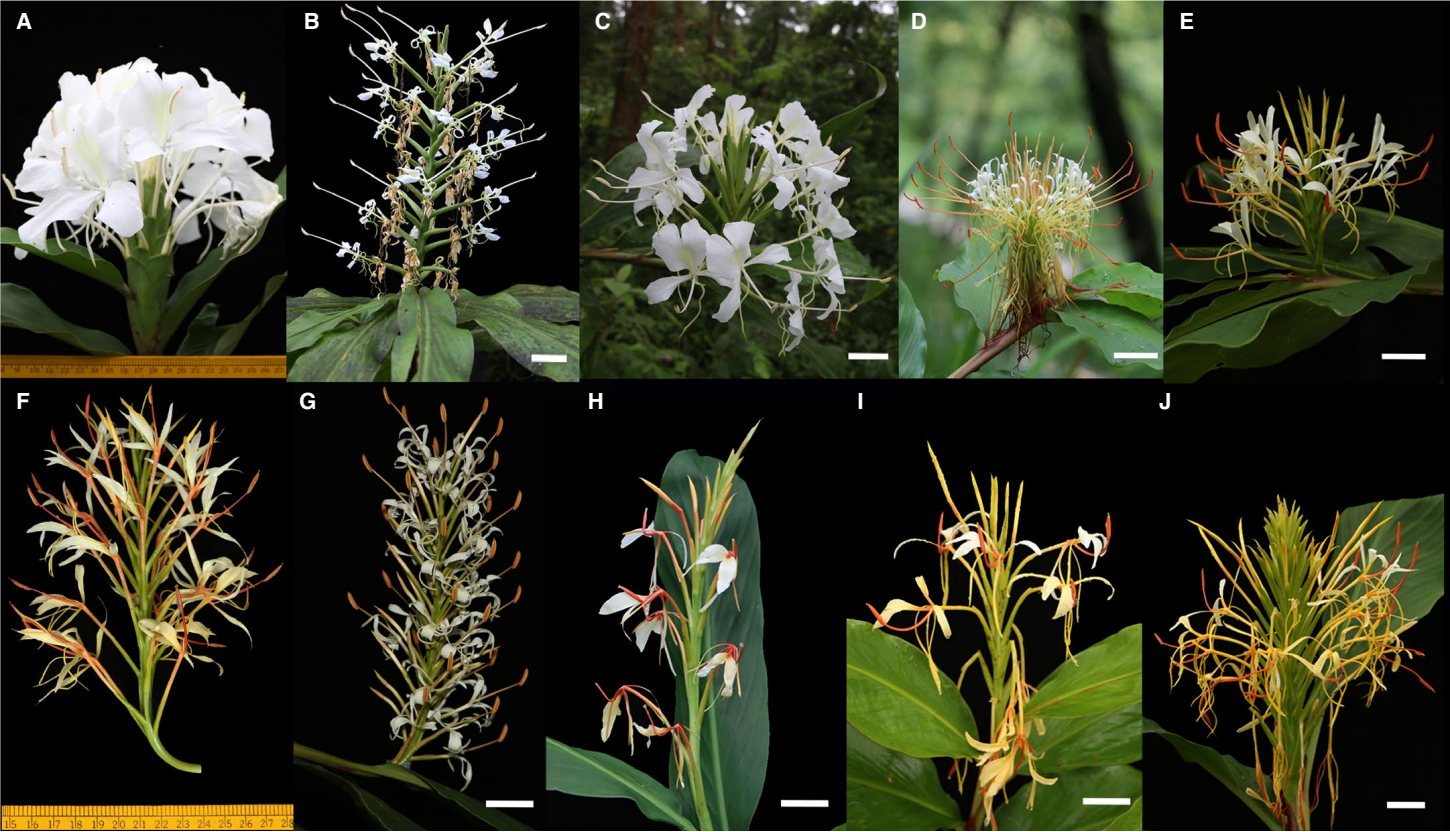
Flowering stalks showing the floral characters in the two species complexes in the genus *Hedychium*. The Coronarium complex consists of *H. coronarium* (A), *H. stenopetalum* (B), and *H. forrestii* (C), while the Spicatum complex consists of *H. ellipticum* (D), *H. gomezianum* (E), *H. gracile* (F), *H. griffithianum* (G), *H. spicatum* (H), *H. spicatum* var. *khasianum* (I), and the “Nongstoin” intermediate form (J). Scale bar = 3 cm. Photos by P. Saryan.

The genus *Hedychium* (~110 species, Zingiberaceae) consists of rhizomatous perennial plants that are known for their fragrant and showy beautiful flowers. The genus is native to the Indian subcontinent, China, and Southeast Asia, and its eastern limit is the Philippines. Taxonomically, *Hedychium* is a difficult genus because of the presence of several species complexes. In this study, we analyzed two species complexes composed of a total of 10 taxa (eight recognized species, one varietal form, and an intermediate form). The two species complexes are: Coronarium complex: *H. coronarium* J. Koenig, *H. forrestii* Diels, and *H. stenopetalum* G. Lodd.; and the Spicatum complex: *H. ellipticum* Buch.‐Ham. ex Sm., *H. gracile* Roxb., *H. griffithianum* Wall., *H. gomezianum* Wall., *H. spicatum* Buch.‐Ham. ex Sm., a varietal form *H. spicatum* var. *khasianum*, and an intermediate form “Nongstoin” (Fig. 1). For detailed species descriptions and their taxonomic and morphological history, refer to Ashokan and Gowda (2017). The morphological data set used for clustering included 12 vegetative and 42 reproductive features (31 quantitative and 23 qualitative characters) quantified from fresh samples collected from wild populations. A minimum of four and a maximum of 19 individuals were measured for all the taxa (*N* = 93 individuals from a total of 10 taxa) included in this study.

### Statistical and machine learning analyses

#### PCA and nMDS

To detect any correlation among the morphological characters, we first performed a Pearson’s correlation test on the quantitative data set using R (version 3.5.2; R Core Team, 2018). Using the FactoMineR package (version 1.41; Lê et al., 2008), PCA was performed on the covariance matrix of mean normalized morphological data from the 10 taxa. To observe the emerging patterns, we visualized the data using character scores from PC1 and PC2. We also performed an nMDS analysis with Gower distances using the VEGAN package version 2.5‐3 in R (Jari et al., 2018). Finally, the first two dimensions (in order of significance of the dimensions for both PCA and nMDS) were passed to the *k*‐means clustering algorithm as inputs to cluster the data in an unsupervised procedure.

#### Machine learning and spectral clustering

The spectral clustering algorithm was implemented in Python version 3.7.4 (Python Software Foundation, 2020). Once clusters were identified, they were validated and used for further character analyses in NumPy version 1.16.4 (Oliphant, 2006), scikit‐learn version 0.20.3 (Pedregosa et al., 2011), and SciPy version 1.2.1 (Virtanen et al., 2020). For data visualization, we used scikit‐learn’s implementation of t‐SNE (van der Maaten and Hinton, 2008). As t‐SNE performs a non‐linear projection of data, it often results in a better visualization as compared to other dimensionality reduction methods. However, points that are seemingly far away in the t‐SNE plot may still be grouped together by spectral clustering as the two methods are independent of each other.

To represent all morphological characters on the same scale, we normalized the data using the mean for each character and then scaled each using the standard deviation in the measurements of that character. In the spectral clustering algorithm, we used a similarity matrix, **S**, which quantified the pairwise similarity between all samples in the data set, and we added *K*, which represented the number of clusters to be discovered. The expected output was an assignment of each sample to one of the discovered clusters. We used a similarity measure, called the radial basis function (RBF) kernel, to compute the entries of matrix **S** as:

$$S_{\mathrm{ij}}=\exp\left(‐\frac{r}{d}{∥x^{\left(i\right)}‐x^{\left(j\right)}∥}^{2}\right)$$

Here, γ is a tuning parameter that is specified by the user and *d* is the number of morphological characters. A larger γ would amplify even small differences between the samples, hence making them more dissimilar, which would lead to many small but more cohesive clusters. We experimented with different values of γ in the range [0.05, 1.0] in increments of 0.05 to test whether the method is sensitive to the choice of this hyper‐parameter. For each value of γ, we used spectral clustering to partition the data into *K* clusters, where *K* was varied from *K*_min_ = 2 to *K*_max_ = 20. *K*_max_ was chosen to be higher than the total number of species and total number of populations, and thus represents the highest number of clusters that may be biologically realized if each population was indeed a true distinct species. We used a normalized symmetric graph Laplacian matrix to perform the spectral clustering. This matrix is given by:

$$L=I‐D^{‐1/2}{\mathrm{SD}}^{‐1/2}$$

Here, the entries of diagonal matrix **D**, for i = 1, 2, …, *N* are computed as:

$$D_{\mathrm{ii}}=\sum_{j=1}^{N}S_{\text{ij}}$$

In summary, each of the *N* data points (here *N* = 93) in the spectral clustering were represented as a d‐dimensional vector that encodes one observed morphological character per dimension. These vectors were grouped into clusters using a spectral clustering algorithm (von Luxburg, 2007), wherein individuals within each cluster were morphologically similar to each other but those in different clusters were different in their morphology. Note that spectral clustering may converge to a local optimum as it uses *k*‐means as a subroutine, i.e., independent executions of the algorithm may yield different clusters. A common way to fix this problem is by running the *k*‐means subroutine several times and selecting the output with the lowest objective function value (using the objective function for *k*‐means). We have set this number to 100 in our experiments. The stability of the algorithm may be improved further by increasing this number, albeit at a higher computational cost (Appendix S1).

### Validation of discovered clusters

The goodness of clusters discovered using spectral clustering was quantified using two approaches: (a) by testing the alignment of the discovered clusters with ground truth clusters that were obtained from a manually annotated data set containing data points with species identity labels, and (b) by quantifying a measure of the similarity of samples within and across the discovered clusters in each analysis. In the first approach, we used the well‐known normalized mutual information (NMI) score and Rand index to quantify the overlap between discovered and ground truth clusters. NMI values range from 0 to 1 and Rand index values range from −1 to 1, with higher values indicating a better alignment in both cases. This approach was used only to demonstrate that the clusters are not arbitrary and have a physical significance. The selection of clusters was purely based on the second approach where we computed the eigengap scores, i.e., difference between successive eigenvalues that have been sorted in the ascending order, and plotted them as an eigengap plot. This plot is commonly used for identifying the total number of clusters in spectral clustering as the peaks in this plot correspond to choices of *K* for which the discovered clusters are cohesive (von Luxburg, 2007). Hence, we used this plot to select appropriate values of *K*. These clusters were next examined for their biological relevance based on domain knowledge (i.e., taxonomic expertise of the user).

### Feature selection

After obtaining clusters via spectral clustering, and choosing values of γ and *K* using the second category of cluster validation methods described above, we identified morphological characters from our input data set that can be used to differentiate the identified clusters. To accomplish this task, we (a) analyzed mutual information between the characters and clusters; (b) analyzed the correlations between the characters and eigenvectors used in the spectral clustering; and (c) studied differences in character values within and outside the clusters using a *t*‐test.

#### Mutual information between characters and clusters

Here, we calculated the mutual information between each morphological character and the cluster assignment proposed by spectral clustering. A high score implies that the character contributes a significant amount of information to the identity of the cluster to which a given sample belongs, thus highlighting the importance of that character.

#### Correlation between eigenvectors and character vectors

As spectral clustering uses eigenvectors of the Laplacian matrix **L**, one way to find the relationship between characters and clusters is to identify the alignment between the character vectors and eigenvectors. To quantify this alignment, we computed the Pearson’s correlation coefficient between every character–eigenvector pair. A high correlation (either positive or negative) implies that the character is useful in differentiating the clusters.

#### Differences in character values within and outside clusters

**Figure 2:**
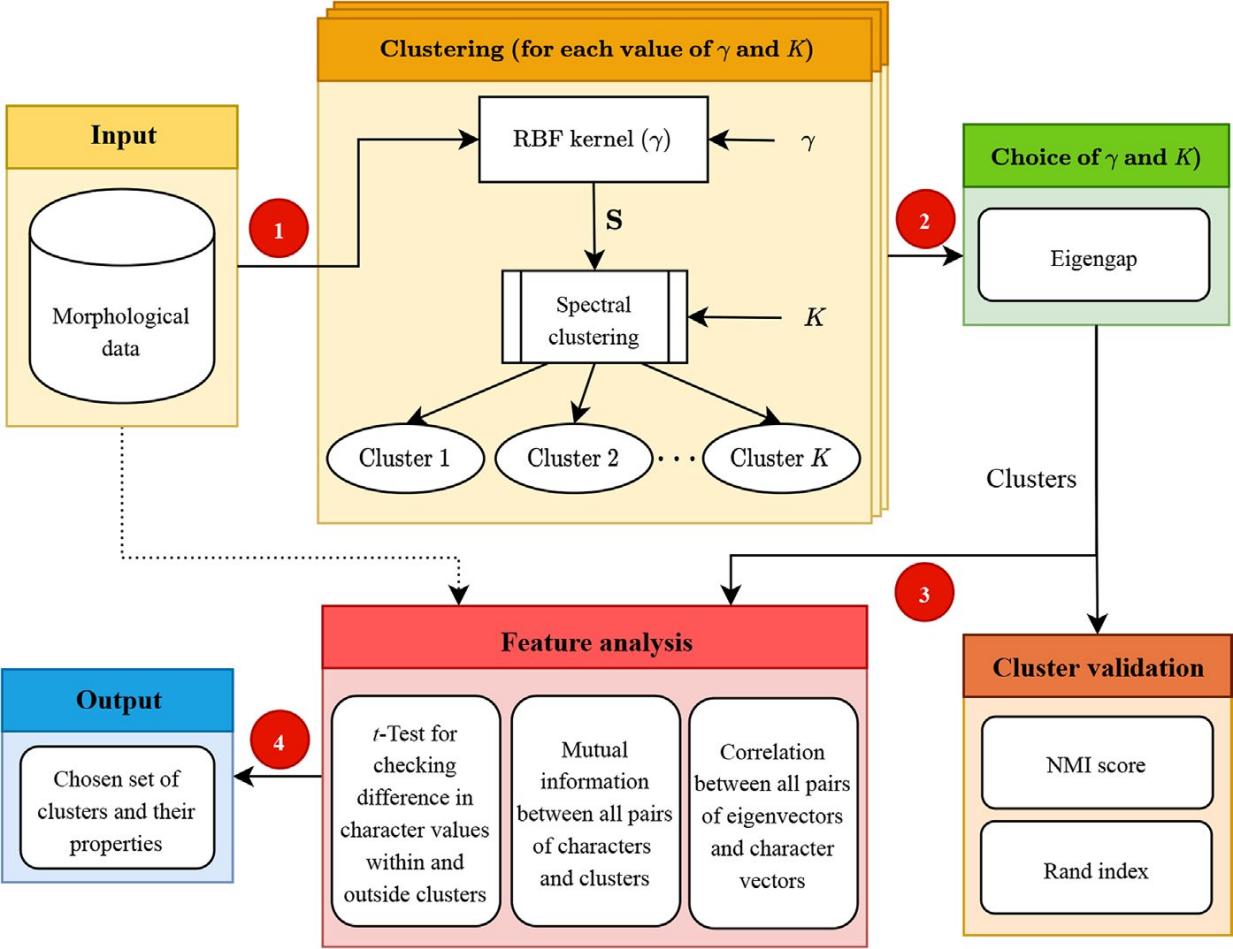
Stepwise representation of the process used to identify clusters and perform feature selection in an unsupervised environment. (1) Morphological data is given as an input, followed by clustering using parameter gamma (γ) for tuning the kernel shape. (2) The eigengap was used to choose γ and the optimal number of clusters. These clusters are then validated using the NMI score and Rand index. (3) The selected clusters along with the raw morphological data (represented by the dotted line) are then subjected to a character analysis. (4) The final output will include the chosen set of clusters and their defining characteristics. NMI = normalized mutual information; RBF = radial basis function; *K* = number of clusters to be discovered; S = pairwise similarity between all samples in the data set.

For each character–cluster pair, we performed a *t*‐test to determine whether the values for the character differ significantly within and outside that cluster. A significant difference acts as an indicator of the importance of the character in differentiating this cluster from others. The details of this method have been summarized in Fig. 2.

## RESULTS

### Statistical and machine learning analyses

To minimize pseudoreplication, 22 characters that were represented by both length and width measurements were converted to ratios. To avoid the duplication of characters wherein correlated characters may be unknowingly represented twice in a morphological data set, we first performed a Pearson’s correlation test on the 31 quantitative characters identified. When biologically meaningful correlations were found between two characters, we retained only one of the characters for further analyses. For example, the number of old flowers in an inflorescence was highly correlated with the number of fertile bracts (*r* ^2^ = 0.75, *P* < 0.05); hence, only the number of fertile bracts was retained for further analysis. The final total number of quantitative characters included in all our analyses was 16 (Table 1).

**Table 1:** List of the morphological characters used in current study.

Character name	Label used in Fig. 5
Plant height	P_Height
Leaf length/width	Leaf_R
Inflorescence length/width	Inf_R
Number of flowers opening per day	Inf_YF
Number of fertile bracts	Inf_bract_fert
Bract length/width	BR_R
Calyx length/width	Cal_R
Floral tube length/width	FTL_R
Floral tube orifice	FTO
Corolla lobe length/width	Clob_R
Lateral staminode length/width	LStam_R
Labellum length/width	Lab_R
Notch depth	Notch
Notch to labellum length ratio	Notch Lab_R
Filament length/width	Fil_R
Nectary length/width	Nect_R

#### PCA and nMDS

**Figure 3:**
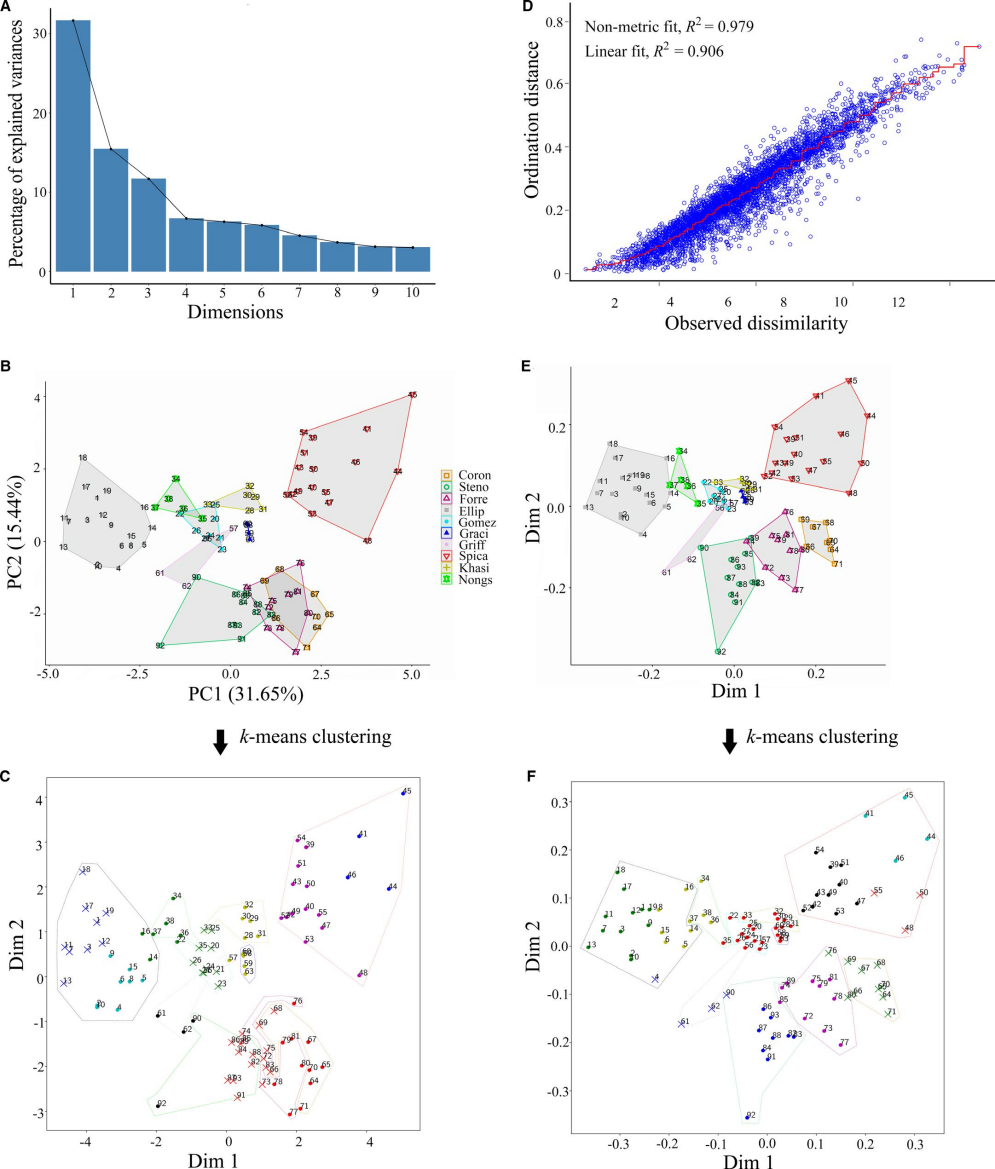
Clusters identified among the two *Hedychium* species complexes using principal component analysis (PCA) and non‐metric multidimensional scaling (nMDS). (A–C) Cluster identification using PCA. (A) Scree plot showing 47.09% of the variation being explained by Principal Component (PC) 1 and PC2. (B) Visualization of the relative positions of each individual within the species‐wise clusters formed by plotting PC1 and PC2. (D–F) Cluster identification using nMDS. (D) Shepard plot showing the disagreement between the 2D configuration and the predicted values from the regression, plotted as observed dissimilarity and ordination distances of 100 iterations in the nMDS analysis. (E) Visualization of the relative position of each individual within the species‐wise cluster formed by plotting the first two dimensions. Dots of the same color in B and E refer to the same species. Outputs of *k*‐means clustering (10 clusters) using the first two dimensions of the PCA and nMDS are presented in C and F, respectively, where the colors represent the cluster assignment. The outlines were drawn manually to represent the ground truth (color representation is same as the legend used in B and E). Coron = *H. coronarium*; Steno = *H. stenopetalum*; Forre = *H. forrestii*; Ellip = *H. ellipticum*; Gomez = *H. gomezianum*; Graci = *H. gracile*; Griff = *H. griffithianum*; Spica = *H. spicatum*; Khasi = *H. spicatum* var. *khasianum*; Nongs = intermediate form Nongstoin.

We first present results from the PCA and nMDS analyses in the form that is traditionally used by biologists (i.e., a visualization) for the species that comprise the morphological species complexes. Next, we present results from the analyses where *k*‐means clustering was performed on the PCA and nMDS outputs. In the PCA, the first four principal components explained 64% of variation in the data (Fig. 3A). The PCA failed to resolve the Coronarium complex as most *H. forrestii* individuals were grouped with *H. coronarium*, and a few showed overlap with *H. stenopetalum* (Fig. 3B). In the Spicatum complex, *H. gomezianum*, *H. griffithianum*, and Nongstoin showed some overlap with each other, with some individuals being grouped into the wrong clusters, leading to ambiguity in species delimitations. *Hedychium ellipticum*, *H. gracile*, and *H. spicatum* formed separate clusters of their own.

The stress value, which represents the agreement between the points in reduced dimensions and predicted values, for the nMDS analysis was 0.17 (Fig. 3D). Stress less than 0.2 indicates good representation of data in reduced dimensions. Contrary to the PCA, the Coronarium complex in the nMDS analysis showed better resolution among the three taxa, with only one *H. forrestii* point overlapping with *H. stenopetalum* (Fig. 3E). In the Spicatum complex, only *H. gomezianum* and *H. griffithianum* showed some overlap, while *H. ellipticum*, *H. gracile*, *H. spicatum* var. *khasianum*, Nongstoin, and *H. spicatum* formed a single separate cluster of their own (Fig. 3E).

When *k*‐means clustering was performed on the first two principal components using the value of *K* = 10, we identified two clusters in the Coronarium complex: the first cluster consisted of *H. coronarium* and *H. forrestii*, and the second cluster consisted of *H. coronarium*, *H. forrestii*, and *H. stenopetalum* (from right to left in Fig. 3C). The Spicatum complex resulted in a total of seven clusters, of which two clusters consisted only of *H. ellipticum* individuals; two clusters comprised only *H. spicatum* individuals; one cluster consisted of individuals from Nongstoin and *H. ellipticum*; one cluster of Nongstoin and *H. gomezianum*; whereas *H. gracile*, *H. griffithianum*, and *H. spicatum* var. *khasianum* together formed a single separate cluster (Fig. 3C). We identified one cluster that acted as an inter‐complex hybrid cluster, which comprised two *H. stenopetalum* and two *H. griffithianum* individuals; this was considered to be an outlier cluster because it is taxonomically irrelevant.

When *k*‐means clustering was performed on the first two dimensions of nMDS with the value of fixed *K* = 10, the Coronarium complex was separated into three clusters: the first cluster consisted of individuals from *H. coronarium* and *H. forrestii*; the second cluster consisted of *H. forrestii* and *H. stenopetalum*; and the third consisted only of *H. stenopetalum* individuals. The Spicatum complex consisted of a total of seven clusters as follows: one cluster of individuals only from *H. ellipticum*; three separate clusters composed only of *H. spicatum* individuals; a cluster formed by Nongstoin with *H. ellipticum*; a single cluster of individuals of *H. gomezianum*, *H. gracile*, *H. griffithianum*, and *H. spicatum* var. *khasianum*; and a cluster containing individuals from *H. ellipticum*, *H. griffithianum*, and *H. stenopetalum* (Fig. 3F).

#### Spectral clustering and validation of discovered clusters

**Figure 4:**
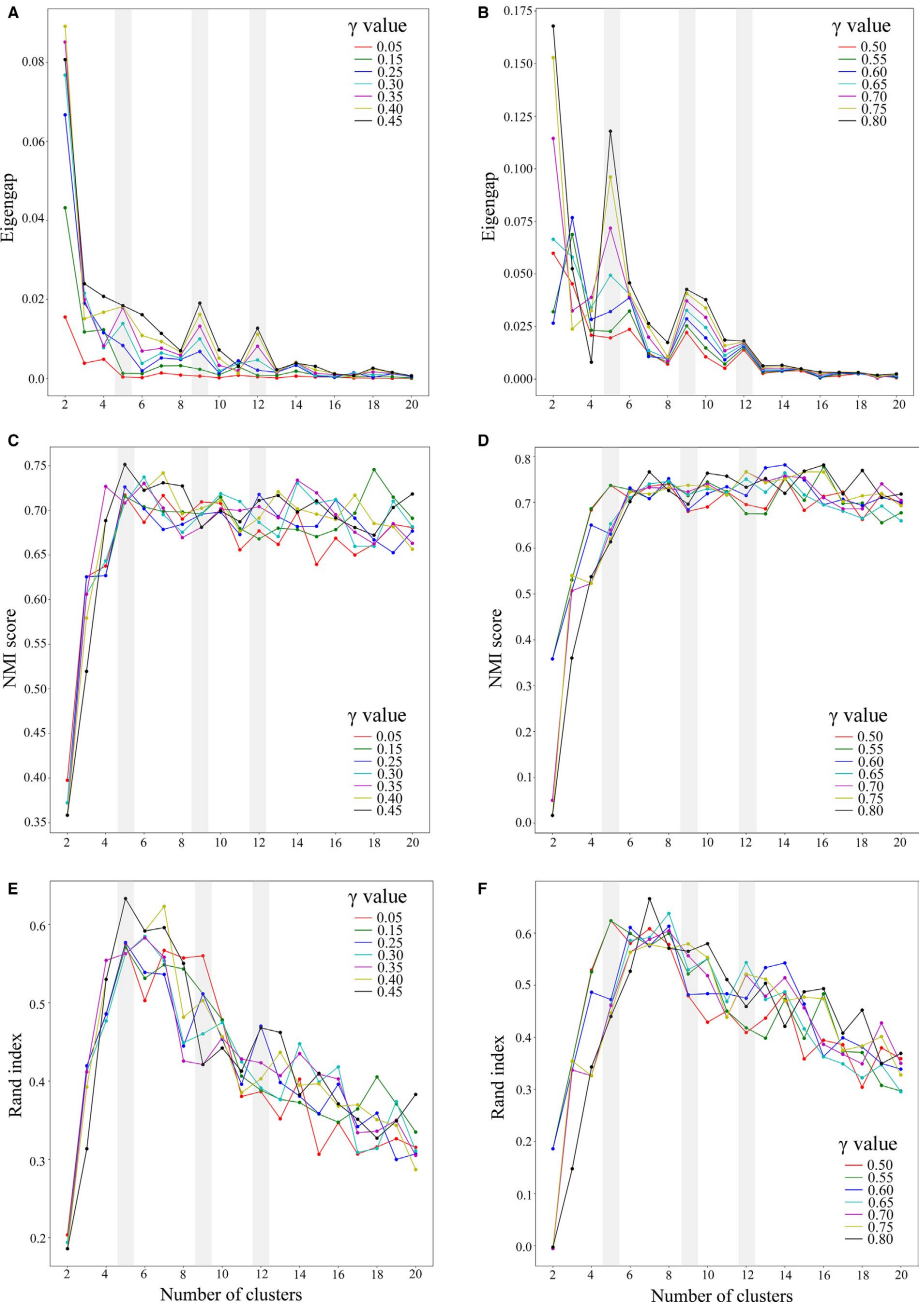
Identification of cluster sizes and their validation for gamma (γ) values. (A, B) Identification of cluster sizes using eigengap. (C, D) Cluster validation using the normalized mutual information (NMI) score. (E, F) Cluster validation using the Rand index. The γ values ranged from 0.05 to 1, and were increased by an increment of 0.05. The gray bars highlight the cluster sizes identified (five, nine, and 12) by considering the eigengap values alone. Because the γ and ***K*** values were chosen based only on eigengap, our method is completely unsupervised.

We observed that most of the γ values resulted in peaks at *K* = 5, 9, and 12, with the eigengap increasing with greater γ values (gray bars in Fig. 4A, B). However, for *K* = 5 (cluster size selected as 5), when γ is greater than 0.50, despite the high eigengap values, species from two different complexes clustered together, which is taxonomically unacceptable and morphologically unlikely. Lower γ values (≤0.30) did not lead to the identification of any prominent clusters, as was evident from the lack of peaks for all values of γ ≤ 0.30 (Fig. 4A, B). Hence, γ values that were ≤0.30 and ≥0.50 were not considered for further analysis, and we were therefore left with γ= 0.35, 0.40, 0.45. The discovered clusters aligned well with our domain expertise in the genus *Hedychium*, which is evident from the high NMI scores (e.g., the NMI score was approximately 0.72 for γ= 0.40 and *K* = 5) and Rand index scores (e.g., the Rand index score was approximately 0.62 for γ = 0.40 and *K* = 5), both of which represent overlap between the discovered and ground truth clusters (Fig. 4C–E). As the resulting clusters were similar across all three chosen values of γ, we present the results for γ = 0.40 only.

**Figure 5:**
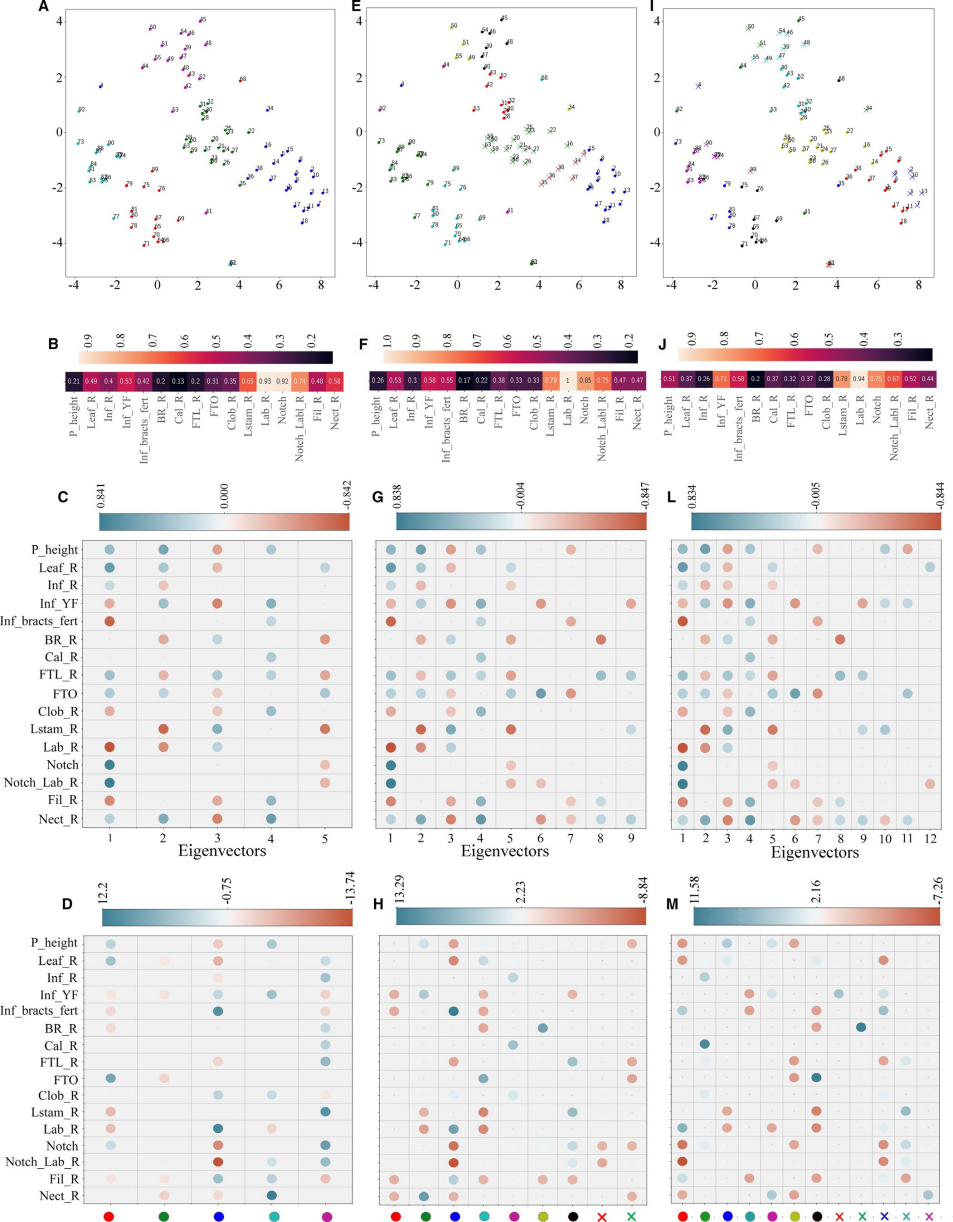
Character analysis of identified clusters. For γ = 0.40, *K* = 5, 9, and 12 clusters (A, E, I, respectively) were subjected to character analysis. From left to right, the columns correspond to *K* = 5, 9, and 12, respectively. The character analysis was performed using mutual information (B, F, J), the correlation of character–eigenvector pair (C, G, L), and *t*‐tests between the selected cluster and other clusters (D, H, M). Correlations of the character–eigenvector pairs are shown in C, G, and L, where the color of the circle represents the Pearson’s correlation coefficient value and the size of the circle represents the significance (larger size represents smaller *P* value [*P* > 0.05 are not shown]). The colors of the dots on the *x*‐axis in D, H, and M correspond to the colors of the clusters given in A, E, and I, respectively. The details of the characters and labels used are provided in Table 1.

When *K* = 5, two clusters were observed in the Coronarium complex and three clusters in the Spicatum complex. When *K* = 9, two clusters were observed in the Coronarium complex and seven clusters in the Spicatum complex. For *K* = 12, we observed four clusters in the Coronarium complex, while the Spicatum complex consisted of six clusters. The composition of these clusters in terms of the placement of individuals from each species in the different clustering analyses (for *K* = 5 to 12) is given in Table 2. We identified few individuals that acted as outliers in all three analyses (i.e., *K* = 5, 9, and 12) due to their placement in cross‐complex clusters or hybrid clusters between two complexes. These individuals are: *H. griffithianum* (#61 and #62); *H. spicatum* (#41, #44, and #45); *H. stenopetalum* (#92); and Nongstoin (#35) (Fig. 5A, E, and I).

**Table 2:** The composition of selected identified clusters (*K* = 5–12) for γ = 0.40 found in the Spicatum complex and Coronarium complex, using the spectral clustering algorithm.

Complex	Clusters	Cluster no.							
		5	6	7	8	9	10	11	12
Spicatum complex	Only *H. ellipticum* individuals	—	—	—	✓	✓	✓	✓	✓
	Only *H. spicatum* individuals	✓	✓	—	✓	✓	✓	—	✓
	*H. ellipticum* + Nongstoin	—	✓	✓	✓	✓	✓	✓	✓
	*H. ellipticum* + *H. griffithianum*	—	—	—	—	—	—	✓	—
	*H. spicatum* var. *khasianum* + *H. spicatum*	—	✓	✓	—	✓	✓	✓	✓
	Nongstoin + *H. spicatum*	—	—	—	—	✓	✓	✓	✓
	*H. ellipticum* + Nongstoin + *H. griffithianum*	✓	—	—	—	—	—	—	—
	*H. gomezianum* + *H. spicatum* var. *khasianum* + *H. spicatum*	—	—	—	✓	—	—	—	—
	*H. gracile* + *H. griffithianum* + *H. gomezianum* + *H. spicatum* var. *khasianum*	—	—	—	—	—	—	✓	—
	*H. gracile* + *H. griffithianum* + *H. gomezianum* + Nongstoin	—	✓	✓	—	—	—	—	—
	*H. ellipticum* + *H. gracile* + *H. griffithianum* + *H. gomezianum* + *H. spicatum* var. *khasianum*	—	—	—	✓	✓	✓	—	✓
	*H. gracile* + *H. griffithianum* + *H. gomezianum* + Nongstoin + *H. spicatum* var. *khasianum*	✓	—	—	—	—	—	—	—
Coronarium complex	Only *H. stenopetalum* individuals	—	—	—	—	—	—	✓	✓
	*H. coronarium* + *H. forrestii*	—	—	✓	—	—	✓	‐	✓
	*H. stenopetalum* + *H. forrestii*	—	—	✓	—	—	✓	✓	✓
	*H. coronarium* + *H. forrestii* + *H. stenopetalum*	✓	✓	—	✓	✓	—	✓	—

### Feature selection

Clusters obtained by setting γ = 0.40 and *K* = 5, 9, and 12 were subjected to character analysis. Our mutual information–based character analysis (Fig. 5B, F, and J) identified the following characters as important: lateral staminode ratio (all characters presented as a ratio represent the length : width of that particular character), labellum ratio, notch, and notch length to labellum length ratio. For *K* = 9 and 12, in addition to the above‐mentioned characters, the number of flowers opening per day and the total number of fertile bracts were also found to be important (Fig. 5F, J, respectively). All of the above characters were also found to be important in the character–eigenvector correlation analysis (Fig. 5C, G, and L). Figures 5C, G, and L represent the first five, nine, and 12 eigenvectors for *K* = 5, 9, and 12, respectively, along with their correlation values (color bar above). Here, the eigenvectors have been placed in decreasing order of their significance in the cluster formation (from left to right). The character that was found to be least important for all clusters (shown in Fig. 5A, E, and I) was the calyx ratio (Fig. 5C, G, and L), whereas all other characters were found to be important in cluster formation.

The results of the *t*‐test analysis for the distribution of characters between a specific cluster and the remaining clusters is given in Fig. 5D, H, and M. Here, the color bar on the top represents the *t* statistics and the color dots on the *x*‐axis represent the clusters identified in Fig. 5A, E, and I for *K* = 5, 9, and 12, respectively. Based on *t*‐test results within the Coronarium complex, for *K* = 5, 9, and 12, the cluster consisting of *H. coronarium* and *H. forrestii* individuals is defined by the floral tube orifice, lateral staminode ratio, and labellum ratio (see red dot in Fig. 5A, cyan dot in 5E, and black dot in 5I, and Fig. 5D, H, and M, respectively, for the *t*‐test statistics). A separate cluster of only *H. forrestii* individuals was observed when *K* = 12, with plant height, lateral staminode ratio, and labellum ratio identified as its defining characters. A separate cluster of only *H. stenopetalum* individuals was also observed at *K* = 12, for which plant height, number of flowers opening per day, labellum ratio, and nectaries ratio were identified as the defining characters (Fig. 5M). In the Spicatum complex, a separate cluster consisting of only *H. spicatum* individuals was observed in *K* = 5, 9, and 12, and lateral staminode ratio, notch length, and filament ratio were identified as its defining characters. A cluster consisting of only *H. ellipticum* individuals was observed for *K* = 9 and 12, with notch length, notch to labellum length ratio, and number of fertile bracts identified as its defining characters. For *K* = 9 and 12, Nongstoin formed a cluster with *H. ellipticum*, for which notch length and notch to labellum ratio were identified as the defining characters. *Hedychium spicatum* var. *khasianum* formed a separate cluster with *H. spicatum* when *K* = 9 and 12, with number of flowers opening per day, number of fertile bracts, and filament ratio identified as the defining characters. *Hedychium gracile*, *H. griffithianum*, and *H. gomezianum* formed a single cluster in *K* = 9 and 12, and plant height, floral tube ratio, floral tube orifice, and nectaries ratio were identified as its defining characters. We also found that for the cross‐complex outlier clusters at *K* = 9 and 12, corolla lobe ratio was identified as the defining character (Fig. 5H, M).

## DISCUSSION

The spectral clustering analyses of the 16 morphological characters resulted in well‐resolved clusters for four species (*H. forrestii*, *H. stenopetalum*, *H. ellipticum*, and *H. spicatum*) and partially resolved clusters for six taxa (*H. coronarium*, *H. gomezianum*, *H. gracile*, *H. griffithianum*, *H. spicatum* var. *khasianum*, and Nongstoin). Within the Coronarium complex, *H. forrestii* and *H. stenopetalum* were confirmed to be distinct, while *H. coronarium* was not. Within the Spicatum complex, *H. ellipticum* and *H. spicatum* were confirmed to be distinct, while the other taxa were not. Our method was found to be efficient and robust in forming clusters in an unsupervised framework and in identifying features that defined the clusters within the chosen species complexes. We propose that (a) clustering is better than manual classification for discovering species groups and hence boundaries; (b) the spectral clustering algorithm is capable of discovering meaningful clusters in complex morphological data; and (c) analyzing the characters of the samples in the discovered clusters can offer biologically relevant insights about the clusters discovered in an unsupervised manner. Together, the spectral clustering algorithm and character analysis methods can be used to understand and analyze the morphological data.

### Comparison of PCA, nMDS, and spectral clustering

A key observation from our study is that the traditional usage of PCA (which is primarily restricted to visualization, as described above) is by itself insufficient for the robust identification of species boundaries in species complexes. Consider, for instance, *H. coronarium*, *H. forrestii*, and *H. stenopetalum*, which are all present in the same geographic area with high morphological variation. Our results showed that nMDS was better able than PCA to differentiate the groups that matched our taxonomic delimitations of the species (e.g., compare the Coronarium complex in Fig. 3B and E). PCA performs a linear projection (Bishop, 2006), whereas nMDS tries to preserve Euclidean distances between the projected samples. As a linear projection might lose critical information in the data, nMDS tends to produce better visualizations than PCA, as was observed in our experiments. Because PCA and nMDS are ordination methods, one must resort to clustering for the unsupervised discovery of clusters. Approaches such as PCA, nMDS, and spectral clustering make use of *k*‐means to cluster data, but differ in the methods by which they obtain the representation of data used by the *k*‐means. The *k*‐means algorithm is a common choice for clustering, and looks for spherical clusters in its input. However, because real‐world data may have non‐spherical clusters as well, the data must first be transformed into an appropriate form suitable for *k*‐means clustering. Whereas PCA and nMDS are primarily dimensionality reduction methods and do not optimize the reduced representation for clustering, spectral clustering performs an eigen‐decomposition of the Laplacian matrix to obtain a reduced representation ideal for clustering. Hence, in practice, spectral clustering yields better clusters.

### Advantages of clustering to characterize species complexes in the genus *Hedychium*

The confusion in species identification could be the result of many factors, such as the absence of discrete characters or insufficient morphological variation for defining a species (for example, in the case of cryptic species), as well as extremely high morphological variation (in the case of hybridization) and/or the taxonomic mislabeling of species. All of the above reasons can result in the designation of a taxonomic species complex. Because it is inherently difficult to assign ground truth labels to taxa within a species complex, the classification‐based approaches (which require ground truth labels) do not provide any independent insights about species boundaries, and it is preferred that an unsupervised clustering method is used instead. For the genus *Hedychium*, we identified at least two species complexes that arise from both taxonomic mislabeling and high morphological variability owing to hybridization, making it an excellent study system to explore the morphological species boundaries using advanced machine learning approaches. In light of the deficiencies in PCA and nMDS noted above, our proposed machine learning approach offers two major advantages: first, it provides a method for discovering arbitrarily shaped clusters by setting the tunable parameters γ and *K*. For example, we found that for *K* = 5 and 9, *H. forrestii* either formed clusters with *H. stenopetalum* or with *H. coronarium*, but for *K* = 12, a separate cluster was identified that contained only the *H. forrestii* individuals (Fig. 5A, E, and I). Second, machine learning offers interpretable insights about the discovered clusters through character analysis. Our machine learning method allowed us to identify intermediates that clustered with either *H. coronarium* or *H. stenopetalum*, and we could identify the associated characters that differentiated them from the other species within the complex. The *H. forrestii* cluster was defined by plant height, lateral staminode ratio, and labellum ratio, whereas the *H. forrestii* individuals, which formed a cluster with *H. coronarium*, were defined by floral tube orifice. In contrast, the individuals that formed a cluster with *H. stenopetalum* were defined by their nectaries ratio (Fig. 5M).

Another sympatric group of populations that show hybridization is that of *H. ellipticum*, *H. spicatum* var. *khasianum*, and *H. spicatum*, in which *H. spicatum* var. *khasianum* is morphologically more similar to *H. spicatum* than to *H. ellipticum* and all three taxa show continuous variation in traits such as filament size. This leads to a taxonomic dilemma in drawing the species distribution, which is evident when *K* = 9 and 12, where *H. spicatum* var. *khasianum* clusters with *H. spicatum* from the same population (Fig. 5E, I). We found that the *t*‐test also identified the filament ratio as the most important character for differentiating the *H. spicatum* var. *khasianum* and *H. spicatum* clusters from the other clusters.

In our analyses, the mutual information value for *K* = 5 (i.e., smaller total clusters; Fig. 5B) suggested the following characters that delimited the clusters: lateral staminodes, labellum, notch to labellum ratios, and notch. As we explored higher numbers of clusters (i.e., *K* = 9 and 12 [Fig. 5F, J, respectively]), the number of flowers opening per day, an ecologically relevant character, also became important in differentiating the clusters. On the other extreme, the mutual information value for characters such as the bract ratio, calyx ratio, and corolla lobes remained low across clusters, suggesting that they may not play an important role in species delimitation within the genus *Hedychium*. We identified a few individuals that always formed cross‐complex outliers in our analyses and, using character analysis, we identified the corolla lobe ratio to be their defining character. Thus, machine learning approaches such as the one presented here allow us not only to discover clusters and resolve a species complex in an unsupervised framework, but also to explore the contribution of different characters toward the identification of the cluster in great detail.

Finally, we observed that species such as *H. gomezianum*, *H. gracile*, *H. griffithianum*, as well as other intermediates, always formed coalesced clusters. We suspect that, due to high levels of hybridization and population variation in their morphology, the currently accepted delimitations may need revision, which should be aided by karyotyping and population genetic studies. Apart from molecular data, information regarding the habitat, sympatry, and overall morphological variability of a species across its populations may also be very useful for differentiating species‐ and/or population‐level clusters. This is where spectral clustering stands out, as it can use an appropriate similarity matrix encoding all of this information from diverse sources to yield a coherent output.

### Concluding remarks, future direction, and broad applicability of our method

The aim of our study is to provide biologists with a method that allows the use of a clustering approach for species delimitation, and that can help them to understand the effect of morphological characters in the formation of clusters. The purpose of the proposed method is not to replace a taxonomist but to validate what experienced taxonomists (who are an “endangered breed”) may infer, and to provide empirical data to support their conclusions. In the current study, by incorporating multifaceted data from ecology (e.g., flowering phenology and sympatric species) and morphology, we were able to elucidate two species complexes within the genus *Hedychium*. The aim of the proposed spectral clustering approach is to allow researchers to explore their data in relation to the features used in the analyses. Our results show that the historical taxonomic groups are supported by eigengap values (which were chosen based on their absolute values), which validates the empirical bases of the taxonomic clusters as chosen by an experienced taxonomist. This was also the inspiration behind not entirely automating the algorithm, because we strongly believe that domain knowledge (i.e., taxonomic understanding of the group) is also critical for a researcher to determine taxonomic boundaries. This will help us avoid the effect of convergence in characters among unrelated taxa on the assignment of taxonomic boundaries.

As an alternative to discovering clusters using manually collected morphological data, one can instead use unprocessed plant images as the input. However, unlike our method, this approach is not interpretable due to the difficulty in identifying the relative importance of various characters. Unlike the *k*‐means clustering algorithm, which is restricted to continuous characters and Euclidean distances, spectral clustering can be applied to many domains, such as biodiversity studies, ecological studies, and genetic and genomic analyses, as it is compatible with any data set (continuous, ordinal, or categorical) from which a similarity matrix can be derived.

The correct identification of species is an indispensable exercise not only due to its taxonomic and evolutionary implications, for example in our understanding of speciation processes, but also because it has direct implications in species counts and therefore biodiversity assessments and the conservation status of a region. Delimiting taxonomic boundaries has always been a heavily debated field because they can be influenced by both the genetic and the environmental components acting on a species (Coyne and Orr, 2004; Wheeler and Meier, 2000; Fujita et al., 2012), and convergence and parallelism can result in similar morphologies from unrelated taxa (Ridley, 2004; Futuyma, 2013). We believe that unsupervised machine learning methods combined with domain knowledge will help taxonomists to make informed decisions about taxonomic boundaries and their identifications. This will, in turn, allow us to make a better assessment of biodiversity and can also aid in identification of variant forms or new species, and zones of active speciation and hybridization, even by untrained eyes. Ultimately, we hope that species assessments from automated machine learning and spectral clustering tools will help land managers and policy makers by allowing them to better quantify and understand the biodiversity within a region, so they can tailor their conservation strategies.

## AUTHOR CONTRIBUTIONS

V.G. conceived the idea, supervised the analysis, and edited the manuscript. P.S. performed the data acquisition. S.G. developed the machine learning framework and the package. P.S. and S.G. performed the statistical analysis and wrote the manuscript. All authors have read and approved the manuscript.

## Supporting information


**APPENDIX S1.** Dependence of standard deviation in the value of *k*‐means objective function on the number of *k*‐means executions.

## Data Availability

This article is part of a larger study that is still in preparation. Codes will be made available as a Python package when the full study is published. Please contact the corresponding author for updated information.
