# Supplementary material for: Species complex delimitations in the genus Hedychium: A machine learning approach for cluster discovery
Source: Appl Plant Sci. 2020 Jul 31;8(7):e11377. doi: 10.1002/aps3.11377 (PMC7394710; doi:10.1002/aps3.11377)

DOI 10.1002/aps3.11377

APPENDIX S1. Dependence of standard deviation in the value of *k*-means objective function on the number of *k*-means executions.

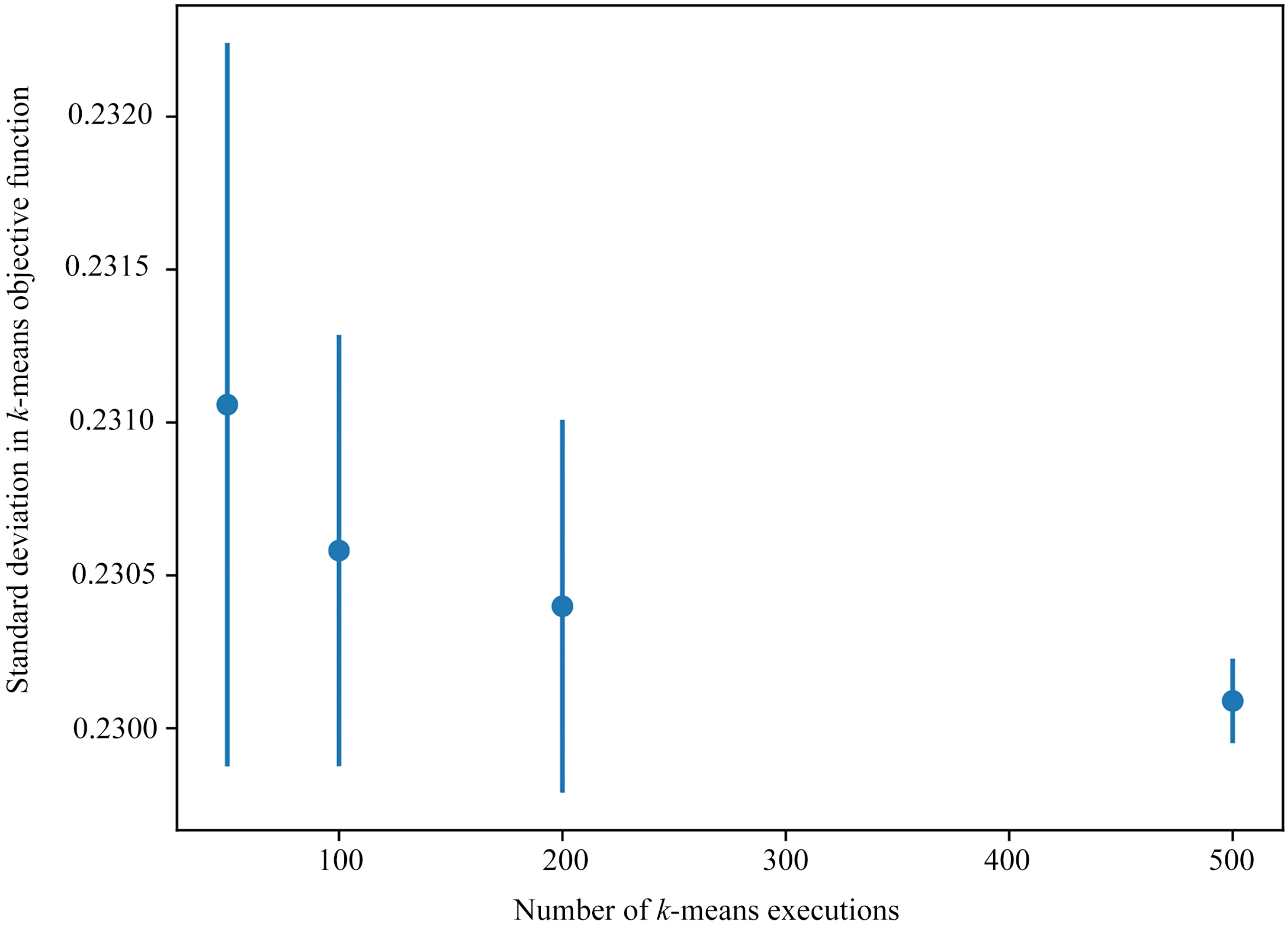

Supplement: Supplementary file 1 — APPENDIX S1. Dependence of standard deviation in the value of k‐means objective function on the number of k‐means executions. [file APS3-8-e11377-s001.pdf]
